# Supplementary material for: Tet2 Controls the Responses of β cells to Inflammation in Autoimmune Diabetes
Source: Nat Commun. 2021 Aug 20;12:5074. doi: 10.1038/s41467-021-25367-z (PMC8379260; doi:10.1038/s41467-021-25367-z)
Supplement: Supplementary file 1 — Supplementary Information [file 41467_2021_25367_MOESM1_ESM.pdf]

Supplementary Table 1: Human islets used in the study

**Islet preparation**	**1**	**2**	**3**	**4**	**5**
Donor age (years)	56	51	31	41	32
Donor sex (M/F)	M	M	M	F	M
Donor BMI (kg/m^2)	32.9	20.3	23.9	18.3	26.2
Donor HbA$_{1c}$	5.30%	5.20%	5.40%	5.50%	4.90%
Origin/source of islets	Prodo Labs	Prodo Labs	Prodo Labs	Prodo Labs	Prodo Labs
Islets isolation centre	Prodo Labs	Prodo Labs	Prodo Labs	Prodo Labs	Prodo Labs
Donor cause of death	Head trauma	Stroke	Head trauma	Stroke	Head trauma
Donor history of diabetes? Yes/No	No	No	No	No	No
Purity by count (%)	90	90	90	90	90-95
Islet viability (%)	95	95	95	95	95

Rank	Motif Name	Consensus	P-value	Log P-value	q-value (Benjamini)
1	CTCF(Zf)/CD4+-CTCF-ChIP-Seq(Barski_et_al.)/Homer	AYAGTGCCMYCTRGTGGCCA	1.00E-05	-1.33E+01	0.0006
2	Fox:Ebox(Forkhead,bHLH)/Panc1-Foxa2-ChIP-Seq(GSE47459)/Homer	NNNVCTGWGYAAACASN	1.00E-05	-1.15E+01	0.0018
3	FOXA1(Forkhead)/MCF7-FOXA1-ChIP-Seq(GSE26831)/Homer	WAAGTAAACA	1.00E-04	-1.01E+01	0.0052
4	Foxa3(Forkhead)/Liver-Foxa3-ChIP-Seq(GSE77670)/Homer	BSNTGTTTACWYWGN	1.00E-04	-9.92E+00	0.0052
5	FOXM1(Forkhead)/MCF7-FOXM1-ChIP-Seq(GSE72977)/Homer	TRTTTACTTW	1.00E-04	-9.52E+00	0.0054
6	FOXA1(Forkhead)/LNCAP-FOXA1-ChIP-Seq(GSE27824)/Homer	WAAGTAAACA	1.00E-03	-9.08E+00	0.0069
7	Pdx1(Homeobox)/Islet-Pdx1-ChIP-Seq(SRA008281)/Homer	YCATYAATCA	1.00E-03	-8.34E+00	0.0125
8	BORIS(Zf)/K562-CTCFL-ChIP-Seq(GSE32465)/Homer	CNNBRGCGCCCCCTGSTGGC	1.00E-03	-8.27E+00	0.0125
9	Foxo3(Forkhead)/U2OS-Foxo3-ChIP-Seq(E-MTAB-2701)/Homer	DGTAAACA	1.00E-03	-8.25E+00	0.0125
10	X-box(HTH)/NPC-H3K4me1-ChIP-Seq(GSE16256)/Homer	GGTTGCCATGGCAA	1.00E-03	-8.06E+00	0.0125
11	Foxa2(Forkhead)/Liver-Foxa2-ChIP-Seq(GSE25694)/Homer	CYTGTTTACWYW	1.00E-03	-7.82E+00	0.0133
12	Oct6(POU,Homeobox)/NPC-Pou3f1-ChIP-Seq(GSE35496)/Homer	WATGCAAATGAG	1.00E-03	-7.53E+00	0.0163
13	Brn1(POU,Homeobox)/NPC-Brn1-ChIP-Seq(GSE35496)/Homer	TATGCWAATBAV	1.00E-03	-7.11E+00	0.0229
14	Rfx1(HTH)/NPC-H3K4me1-ChIP-Seq(GSE16256)/Homer	KGTTGCCATGGCAA	1.00E-02	-6.27E+00	0.0492
15	Lhx3(Homeobox)/Neuron-Lhx3-ChIP-Seq(GSE31456)/Homer	ADBTAATTAR	1.00E-02	-6.26E+00	0.0492

Supplementary Table 2. Homer motif analysis of the transcription factors that potentially bind the chromatin sites with increased accessibility in β cells from KO recipients. See **ATAC-seq and Data Analysis** in the methods section for the details of data analysis.

Supplementary Table 3. Homer motif analysis of the transcription factors that potentially bind the chromatin sites with reduced accessibility in β cells from KO recipients.

Rank	Motif Name	Consensus	P-value	Log P-value	q-value (Benjamini)
1	IRF2(IRF)/Erythroblas-IRF2-ChIP-Seq(GSE36985)/Homer	GAAASYGAAASY	1.00E-17	-3.92E+01	0
2	Foxa3(Forkhead)/Liver-Foxa3-ChIP-Seq(GSE77670)/Homer	BSNTGTTTACWYWGN	1.00E-16	-3.87E+01	0
3	Foxa2(Forkhead)/Liver-Foxa2-ChIP-Seq(GSE25694)/Homer	CYTGTTTACWYW	1.00E-14	-3.31E+01	0
4	IRF1(IRF)/PBMC-IRF1-ChIP-Seq(GSE43036)/Homer	GAAAGTGAAAGT	1.00E-13	-3.09E+01	0
5	Nkx6.1(Homeobox)/Islet-Nkx6.1-ChIP-Seq(GSE40975)/Homer	GKTAATGR	1.00E-12	-2.77E+01	0
6	Fox:Ebox(Forkhead,bHLH)/Panc1-Foxa2-ChIP-Seq(GSE47459)/Homer	NNNVCTGWGYAAACASN	1.00E-11	-2.62E+01	0
7	FOXM1(Forkhead)/MCF7-FOXM1-ChIP-Seq(GSE72977)/Homer	TRTTTACTTW	1.00E-11	-2.57E+01	0
8	ISRE(IRF)/ThioMac-LPS-Expression(GSE23622)/Homer	AGTTTCASTTTC	1.00E-11	-2.56E+01	0
9	Lhx2(Homeobox)/HFSC-Lhx2-ChIP-Seq(GSE48068)/Homer	TAATTAGN	1.00E-10	-2.49E+01	0
10	FOXA1(Forkhead)/MCF7-FOXA1-ChIP-Seq(GSE26831)/Homer	WAAGTAAACA	1.00E-10	-2.41E+01	0
11	FOXA1(Forkhead)/LNCAP-FOXA1-ChIP-Seq(GSE27824)/Homer	WAAGTAAACA	1.00E-09	-2.28E+01	0
12	Lhx3(Homeobox)/Neuron-Lhx3-ChIP-Seq(GSE31456)/Homer	ADBTAATTAR	1.00E-09	-2.25E+01	0
13	Lhx1(Homeobox)/EmbryoCarcinoma-Lhx1-ChIP-Seq(GSE70957)/Homer	NNYTAATTAR	1.00E-09	-2.13E+01	0
14	Foxo1(Forkhead)/RAW-Foxo1-ChIP-Seq(Fan_et_al.)/Homer	CTGTTTAC	1.00E-08	-2.00E+01	0
15	IRF8(IRF)/BMDM-IRF8-ChIP-Seq(GSE77884)/Homer	GRAASTGAAAST	1.00E-08	-1.93E+01	0
16	IRF3(IRF)/BMDM-Irf3-ChIP-Seq(GSE67343)/Homer	AGTTTCAKTTTC	1.00E-08	-1.88E+01	0
17	FOXK2(Forkhead)/U2OS-FOXK2-ChIP-Seq(E-MTAB-2204)/Homer	SCHTGTTTACAT	1.00E-07	-1.75E+01	0
18	Isl1(Homeobox)/Neuron-Isl1-ChIP-Seq(GSE31456)/Homer	CTAATKGV	1.00E-07	-1.69E+01	0
19	FOXK1(Forkhead)/HEK293-FOXK1-ChIP-Seq(GSE51673)/Homer	NVWTGTTTAC	1.00E-07	-1.69E+01	0
20	Foxo3(Forkhead)/U2OS-Foxo3-ChIP-Seq(E-MTAB-2701)/Homer	DGTAAACA	1.00E-07	-1.67E+01	0
21	NeuroG2(bHLH)/Fibroblast-NeuroG2-ChIP-Seq(GSE75910)/Homer	ACCATCTGTT	1.00E-06	-1.58E+01	0
22	Fra2(bZIP)/Striatum-Fra2-ChIP-Seq(GSE43429)/Homer	GGATGACTCATC	1.00E-06	-1.57E+01	0
23	NeuroD1(bHLH)/Islet-NeuroD1-ChIP-Seq(GSE30298)/Homer	GCCATCTGTT	1.00E-06	-1.56E+01	0
24	Stat3(Stat)/mES-Stat3-ChIP-Seq(GSE11431)/Homer	CTTCCGGGAA	1.00E-06	-1.55E+01	0
25	Fra1(bZIP)/BT549-Fra1-ChIP-Seq(GSE46166)/Homer	NNATGASTCATH	1.00E-06	-1.52E+01	0
26	Six1(Homeobox)/Myoblast-Six1-ChIP-Chip(GSE20150)/Homer	GKVTCADRTTWC	1.00E-06	-1.48E+01	0
27	Nanog(Homeobox)/mES-Nanog-ChIP-Seq(GSE11724)/Homer	RGCCATTAAC	1.00E-06	-1.48E+01	0
28	SCL(bHLH)/HPC7-Scl-ChIP-Seq(GSE13511)/Homer	AVCAGCTG	1.00E-06	-1.47E+01	0
29	Tcf21(bHLH)/ArterySmoothMuscle-Tcf21-ChIP-Seq(GSE61369)/Homer	NAACAGCTGG	1.00E-06	-1.43E+01	0
30	Atoh1(bHLH)/Cerebellum-Atoh1-ChIP-Seq(GSE22111)/Homer	VNRVCAGCTGGY	1.00E-05	-1.37E+01	0
31	Pdx1(Homeobox)/Islet-Pdx1-ChIP-Seq(SRA008281)/Homer	YCATYAATCA	1.00E-05	-1.36E+01	0
32	AP-1(bZIP)/ThioMac-PU.1-ChIP-Seq(GSE21512)/Homer	VTGACTCATC	1.00E-05	-1.36E+01	0
33	FoxL2(Forkhead)/Ovary-FoxL2-ChIP-Seq(GSE60858)/Homer	WWTRTAAACAVG	1.00E-05	-1.36E+01	0
34	Stat3+il21(Stat)/CD4-Stat3-ChIP-Seq(GSE19198)/Homer	SVYTTCCNGGAARB	1.00E-05	-1.35E+01	0
35	HEB(bHLH)/mES-Heb-ChIP-Seq(GSE53233)/Homer	VCAGCTGBNN	1.00E-05	-1.35E+01	0
36	Rfx6(HTH)/Min6b1-Rfx6.HA-ChIP-Seq(GSE62844)/Homer	TGTTKCCTAGCAACM	1.00E-05	-1.32E+01	0
37	MyoG(bHLH)/C2C12-MyoG-ChIP-Seq(GSE36024)/Homer	AACAGCTG	1.00E-05	-1.31E+01	0
38	JunB(bZIP)/DendriticCells-Junb-ChIP-Seq(GSE36099)/Homer	RATGASTCAT	1.00E-05	-1.31E+01	0
39	FOXP1(Forkhead)/H9-FOXP1-ChIP-Seq(GSE31006)/Homer	NYYTGTTTACHN	1.00E-05	-1.25E+01	0
40	BATF(bZIP)/Th17-BATF-ChIP-Seq(GSE39756)/Homer	DATGASTCAT	1.00E-05	-1.23E+01	0
41	NF1(CTF)/LNCAP-NF1-ChIP-Seq(Unpublished)/Homer	CYTGGCABNSTGCCAR	1.00E-05	-1.23E+01	0
42	Foxf1(Forkhead)/Lung-Foxf1-ChIP-Seq(GSE77951)/Homer	WWATRTAAACAN	1.00E-05	-1.22E+01	0
43	Ap4(bHLH)/AML-Tfap4-ChIP-Seq(GSE45738)/Homer	NAHCAGCTGD	1.00E-05	-1.18E+01	0.0001
44	Ptf1a(bHLH)/Panc1-Ptf1a-ChIP-Seq(GSE47459)/Homer	ACAGCTGTTN	1.00E-05	-1.17E+01	0.0001
45	Rfx5(HTH)/GM12878-Rfx5-ChIP-Seq(GSE31477)/Homer	SCCTAGCAACAG	1.00E-05	-1.17E+01	0.0001
46	Atf3(bZIP)/GBM-ATF3-ChIP-Seq(GSE33912)/Homer	DATGASTCATHN	1.00E-05	-1.17E+01	0.0001
47	Ascl1(bHLH)/NeuralTubes-Ascl1-ChIP-Seq(GSE55840)/Homer	NNVVCAGCTGBN	1.00E-04	-1.13E+01	0.0001
48	NFkB-p65(RHD)/GM12787-p65-ChIP-Seq(GSE19485)/Homer	WGGGGATTTCCC	1.00E-04	-1.05E+01	0.0002
49	Atf7(bZIP)/3T3L1-Atf7-ChIP-Seq(GSE56872)/Homer	NGRTGACGTCAY	1.00E-04	-1.05E+01	0.0002
50	STAT1(Stat)/HelaS3-STAT1-ChIP-Seq(GSE12782)/Homer	NATTTCCNGGAAAT	1.00E-04	-1.02E+01	0.0003
51	Mef2d(MADS)/Retina-Mef2d-ChIP-Seq(GSE61391)/Homer	GCTATTTTTAGC	1.00E-04	-1.01E+01	0.0003
52	E2A(bHLH)/proBcell-E2A-ChIP-Seq(GSE21978)/Homer	DNRCAGCTGY	1.00E-04	-9.97E+00	0.0003
53	IRF4(IRF)/GM12878-IRF4-ChIP-Seq(GSE32465)/Homer	ACTGAAACCA	1.00E-04	-9.92E+00	0.0003
54	Fosl2(bZIP)/3T3L1-Fosl2-ChIP-Seq(GSE56872)/Homer	NATGASTCABNN	1.00E-04	-9.70E+00	0.0004
55	MafA(bZIP)/Islet-MafA-ChIP-Seq(GSE30298)/Homer	TGCTGACTCA	1.00E-04	-9.40E+00	0.0005
56	PRDM1(Zf)/Hela-PRDM1-ChIP-Seq(GSE31477)/Homer	ACTTTCACTTTC	1.00E-03	-9.11E+00	0.0007
57	NFkB-p65-Rel(RHD)/ThioMac-LPS-Expression(GSE23622)/Homer	GGAAATTCCC	1.00E-03	-8.98E+00	0.0008
58	STAT4(Stat)/CD4-Stat4-ChIP-Seq(GSE22104)/Homer	NYTTCCWGGAAR	1.00E-03	-8.89E+00	0.0009
59	BMYB(HTH)/Hela-BMYB-ChIP-Seq(GSE27030)/Homer	NHAACBGYYV	1.00E-03	-8.60E+00	0.0011
60	Tbr1(T-box)/Cortex-Tbr1-ChIP-Seq(GSE71384)/Homer	AAGGTGTKAA	1.00E-03	-8.44E+00	0.0013
61	NF1-halfsite(CTF)/LNCaP-NF1-ChIP-Seq(Unpublished)/Homer	YTGCCAAG	1.00E-03	-8.28E+00	0.0015
62	Atf2(bZIP)/3T3L1-Atf2-ChIP-Seq(GSE56872)/Homer	NRRTGACGTCAT	1.00E-03	-8.23E+00	0.0016
63	PU.1:IRF8(ETS:IRF)/pDC-Irf8-ChIP-Seq(GSE66899)/Homer	GGAAGTGAAAST	1.00E-03	-7.43E+00	0.0034
64	Jun-AP1(bZIP)/K562-cJun-ChIP-Seq(GSE31477)/Homer	GATGASTCATCN	1.00E-03	-7.29E+00	0.0039
65	MyoD(bHLH)/Myotube-MyoD-ChIP-Seq(GSE21614)/Homer	RRCAGCTGYTSY	1.00E-03	-7.14E+00	0.0045
66	Tcf12(bHLH)/GM12878-Tcf12-ChIP-Seq(GSE32465)/Homer	VCAGCTGYTG	1.00E-03	-7.12E+00	0.0045
67	MITF(bHLH)/MastCells-MITF-ChIP-Seq(GSE48085)/Homer	RTCATGTGAC	1.00E-03	-7.02E+00	0.0049
68	Unknown-ESC-element(?)/mES-Nanog-ChIP-Seq(GSE11724)/Homer	CACAGCAGGGGG	1.00E-02	-6.86E+00	0.0056
69	c-Jun-CRE(bZIP)/K562-cJun-ChIP-Seq(GSE31477)/Homer	ATGACGTCATCY	1.00E-02	-6.74E+00	0.0063
70	Hoxb4(Homeobox)/ES-Hoxb4-ChIP-Seq(GSE34014)/Homer	TGATTRATGGCY	1.00E-02	-6.69E+00	0.0064
71	Bach2(bZIP)/OCILy7-Bach2-ChIP-Seq(GSE44420)/Homer	TGCTGAGTCA	1.00E-02	-6.49E+00	0.0078
72	Atf1(bZIP)/K562-ATF1-ChIP-Seq(GSE31477)/Homer	GATGACGTCA	1.00E-02	-6.41E+00	0.0083
73	CRE(bZIP)/Promoter/Homer	CSGTGACGTCAC	1.00E-02	-6.40E+00	0.0083
74	USF1(bHLH)/C2C12-Usf1-ChIP-Seq(GSE36030)/Homer	SGTCACGTGR	1.00E-02	-6.39E+00	0.0083
75	Nur77(NR)/K562-NR4A1-ChIP-Seq(GSE31363)/Homer	TGACCTTTNCNT	1.00E-02	-6.19E+00	0.01
76	Olig2(bHLH)/Neuron-Olig2-ChIP-Seq(GSE30882)/Homer	RCCATMTGTT	1.00E-02	-6.17E+00	0.0101
77	Mef2b(MADS)/HEK293-Mef2b.V5-ChIP-Seq(GSE67450)/Homer	GCTATTTTTGGM	1.00E-02	-6.13E+00	0.0103
78	Usf2(bHLH)/C2C12-Usf2-ChIP-Seq(GSE36030)/Homer	GTCACGTGGT	1.00E-02	-5.71E+00	0.0155
79	HNF1b(Homeobox)/PDAC-HNF1B-ChIP-Seq(GSE64557)/Homer	GTTAATNATTAA	1.00E-02	-5.56E+00	0.0177
80	Tbet(T-box)/CD8-Tbet-ChIP-Seq(GSE33802)/Homer	AGGTGTGAAM	1.00E-02	-5.55E+00	0.0177
81	Tgif2(Homeobox)/mES-Tgif2-ChIP-Seq(GSE55404)/Homer	TGTCANYT	1.00E-02	-5.54E+00	0.0177
82	Tbx5(T-box)/HL1-Tbx5.biotin-ChIP-Seq(GSE21529)/Homer	AGGTGTCA	1.00E-02	-5.45E+00	0.019
83	STAT5(Stat)/mCD4+-Stat5-ChIP-Seq(GSE12346)/Homer	RTTTCTNAGAAA	1.00E-02	-5.41E+00	0.0196
84	Myf5(bHLH)/GM-Myf5-ChIP-Seq(GSE24852)/Homer	BAACAGCTGT	1.00E-02	-5.40E+00	0.0196

85	Fli1(ETS)/CD8-FLI-ChIP-Seq(GSE20898)/Homer	NRYTTCCGGH	1.00E-02	-5.35E+00	0.0203
86	Six2(Homeobox)/NephronProgenitor-Six2-ChIP-Seq(GSE39837)/Homer	GWAAYHTGAKMC	1.00E-02	-5.33E+00	0.0205
87	Esrrb(NR)/mES-Esrrb-ChIP-Seq(GSE11431)/Homer	KTGACCTTGA	1.00E-02	-5.00E+00	0.0282
88	Zic3(Zf)/mES-Zic3-ChIP-Seq(GSE37889)/Homer	GGCCYCCTGCTGDGH	1.00E-02	-5.00E+00	0.0282
89	BORIS(Zf)/K562-CTCFL-ChIP-Seq(GSE32465)/Homer	CNNBRGCGCCCCCTGSTGGC	1.00E-02	-4.91E+00	0.0301
90	Mef2a(MADS)/HL1-Mef2a.biotin-ChIP-Seq(GSE21529)/Homer	CYAAAAATAG	1.00E-02	-4.88E+00	0.0306
91	Srebp1a(bHLH)/HepG2-Srebp1a-ChIP-Seq(GSE31477)/Homer	RTCACSCCAY	1.00E-02	-4.80E+00	0.033
92	c-Myc(bHLH)/LNCAP-cMyc-ChIP-Seq(Unpublished)/Homer	VCCACGTG	1.00E-02	-4.79E+00	0.033
93	HOXA2(Homeobox)/mES-Hoxa2-ChIP-Seq(Donaldson_et_al.)/Homer	GYCATCMATCAT	1.00E-02	-4.76E+00	0.0337
94	Elk4(ETS)/Hela-Elk4-ChIP-Seq(GSE31477)/Homer	NRYTTCCGGY	1.00E-01	-4.57E+00	0.0402
95	CLOCK(bHLH)/Liver-Clock-ChIP-Seq(GSE39860)/Homer	GHCACGTG	1.00E-01	-4.49E+00	0.0432
96	Zic(Zf)/Cerebellum-ZIC1.2-ChIP-Seq(GSE60731)/Homer	CCTGCTGAGH	1.00E-01	-4.38E+00	0.0475
97	IRF:BATF(IRF:bZIP)/pDC-Irf8-ChIP-Seq(GSE66899)/Homer	CTTTCANTATGACTV	1.00E-01	-4.35E+00	0.0483

Supplementary Table 4: Primer pairs used for RT-PCR analysis

	primers used for qRT-PCR analysis of transcription level	
TET2-F	GGCTACAAAGCTCCAGAATGG	
TET2-R	AAGAGTGCCACTTGGTGTCTC	
Tet2-F	AGAGAAGACAATCGAGAAGTCGG	
Tet2-R	CCTTCCGTACTCCCAAACTCAT	
Il6-F	TCTATACCACTTCACAAGTCGGA	
Il6-R	GAATTGCCATTGCACAACTCTTT	
Ccl2-F	TTAAAAACCTGGATCGGAACCAA	
Ccl2-R	GCATTAGCTTCAGATTTACGGGT	
Cxcl10-F	CCAAGTGCTGCCGTCATTTTC	
Cxcl10-R	GGCTCGCAGGGATGATTTCAA	
Cxcl16-F	CCTTGTCTCTTGCGTTCTTCC	
Cxcl16-R	TCCAAAGTACCCTGCGGTATC	
Tnf-F	CAGGCGGTGCCTATGTCTC	
Tnf-R	CGATCACCCCGAAGTTCAGTAG	
Fas-F	GCGGGTTCGTGAAACTGATAA	
Fas-R	GCAAAATGGGCCTCCTTGATA	
Tnfrsf10b-F	CGGGCAGATCACTACACCC	
Tnfrsf10b-R	TGTTACTGGAACAAAGACAGCC	
stat1-F	TCACAGTGGTTCGAGCTTCAG	
stat1-R	CGAGACATCATAGGCAGCGTG	
Ccl5-F	GCTGCTTTGCCTACCTCTCC	
Ccl5-R	TCGAGTGACAAACACGACTGC	
Cxcl12-F	TGCATCAGTGACGGTAAACCA	
Cxcl12-R	TTCTTCAGCCGTGCAACAATC	
Ccl19-F	CCTGGGAACATCGTGAAAGC	
Ccl19-R	TAGTGTGGTGAACACAACAGC	
Ccl20-F	ACTGTTGCCTCTCGTACATACA	
Ccl20-R	GAGGAGGTTCACAGCCCTTTT	
Il1b-F	GAAATGCCACCTTTTGACAGTG	
Il1b-R	TGGATGCTCTCATCAGGACAG	
Cxcl9-F	GGAGTTCGAGGAACCCTAGTG	
Cxcl9-R	GGGATTTGTAGTGGATCGTGC	
Cxcl11-F	GGCTTCCTTATGTTCAAACAGGG	
Cxcl11-R	GCCGTTACTCGGGTAAATTACA	
Pdl1 -F	AGTATGGCAGCAACGTCACG	
Pdl1 -R	TCCTTTTCCCAGTACACCACTA	
Ido-F	TGGCGTATGTGTGGAACCG	
Ido-R	CTCGCAGTAGGGAACAGCAA	
Actb-F	GGCTGTATTCCCCTCCATCG	
Actb-R	CCAGTTGGTAACAATGCCATGT	
	**Primer pairs used for hMeDIP qRT-PCR analysis**	**# of CpG sites**
Il6 -F	GTGTGTGTGTGTGTGTGTATG	5
Il6 -R	GAGAAAGAGAAGCTAAAGCTGATG	
Cxcl16-F	CTGGATCAGTTTGGTGCGAA	10

Cxcl16-R	GTCACGACGGTCCTCCA	
cxcl10-F	CTCACGCTTTGGAAAGTGAAAC	3
cxcl10-R	AGGGCATTGCTTGTGTTTATTG	
Fas-F	CGATTTCTGGGAAGACCTGAATAG	5
Fas-R	TAGACTGCCTGTGGGTATGT	
Stat1-F	TTTCCTGCCCGGTACTTTC	10
Stat1-R	GAGTCTGGGCAAATCTCTCTG	
Ctrl neg-F	GACTTCTGACTTATACACATACATAC	
Ctrl neg-R	CAACCAGTGCTCTTAACAAC	

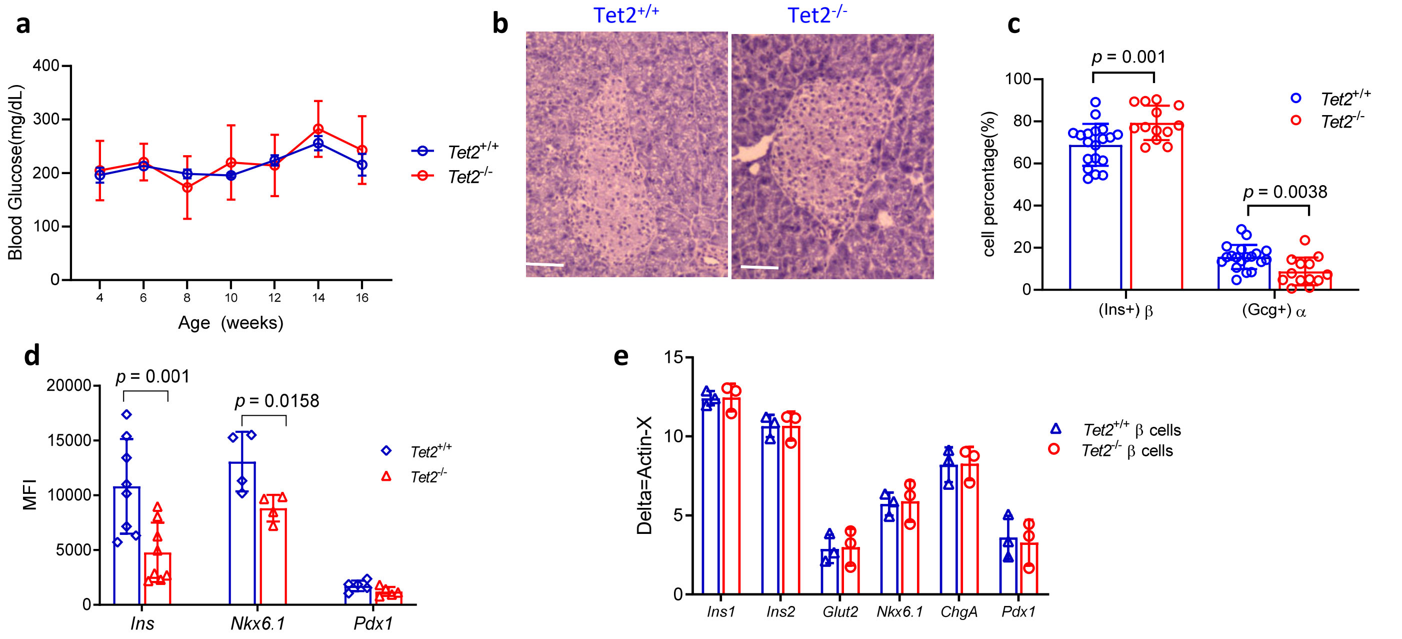

Supplementary Fig 1, related to Fig 3. **Normal glucose tolerance and altered islets composition in Tet2 deficient mice. a** AUC glucose level in Tet2-wild type (Tet2$^{+/+}$) and deficient (Tet2-/-, Tet2$^{-/-}$) mice (B6 background) was measured every two weeks during IPGTTs from 4 weeks to 16 weeks of age (Data are mean ± SD; n=6-8 mice each group). Statistical analysis was performed using two-way ANOVA (repeated measures) with Sidak's multiple comparisons test. **b** H&E staining of the pancreas slides showing one islet from Tet2-Tet2+/+ vs Tet2-/- B6 mice. Data represent at least 3 mice from each group. Scale bars: 25µm. **c** β as well as α cells compartment in islets show as percentage of total islet cells, following intracellular staining with antibodies against insulin and glucagon and FACS analysis. Data (mean ± SD) are from ≥ 5 experiments. Each circle represents a mouse. **d** Median fluorescence intensity (MFI) showing Insulin, Nkx6.1 as well as Pdx1 content in β cells from Tet2+/+ and Tet2-/- mice following intracellular staining and FACS analysis. Data are from ≥ 5 experiments. One circle represents one mouse. **e** Transcriptional analysis of genes that are critical for β cell function and identity in β cells from Tet2-/- vs Tet2+/+ mice. Insulin+ β cells were sorted following intracellular staining and mRNA level was determined after RNA recovery and cDNA synthesis. Data are from 3 individual experiments (mean ± SD), representing 4 sorts, n=4-5 mice each group per sort. Statistical analysis was performed using using two-way ANOVA with Sidak's ((c, e) or Tukey's (d) multiple comparisons test.

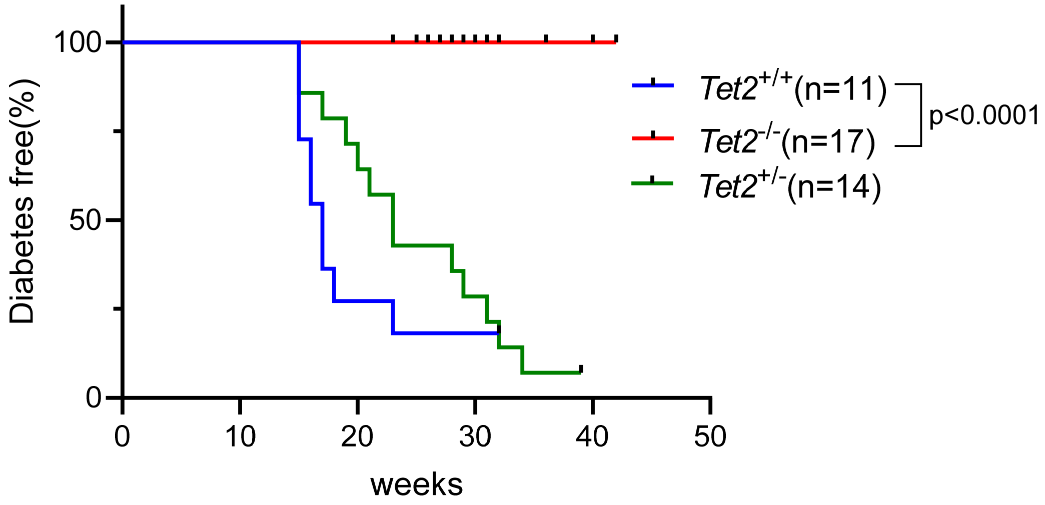

Supplementary Fig 2, related to Fig 3. **Diabetes incidence in Tet2+/+ and Tet2-/- NOD mice**. Tet2+/+ , Tet2+/- and Tet2-/- NOD mice were followed twice weekly for diabetes incidence up to 40 weeks of age. Results are from 11 Tet2+/+, 14 Tet2+/- and 17 Tet2-/- female NOD mice. Statistical analysis was performed using Log-rank curve comparison.

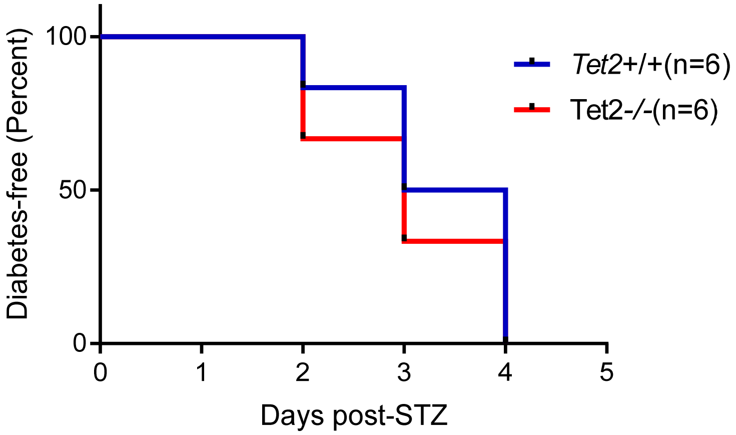

Supplementary Fig 3, related to Fig 3. **β cells from Tet2-/- mice are not protected from streptozotocin killing.** Diabetes incidence in Tet2+/+ and Tet2-/- B6 mice following streptozotocin (STZ) treatment. Mice were given a single dose of STZ (200 mg/kg, i.p) and followed daily for diabetes. Data are from 2 experiments and 3 mice per group per experiment. Statistical analysis was performed using Log-rank curve comparison.

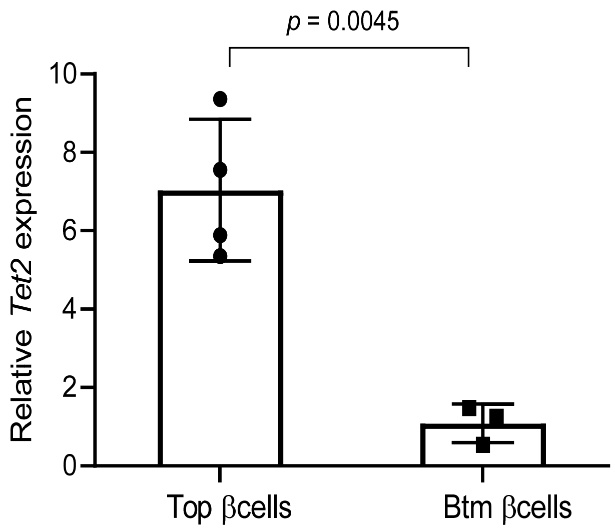

Supplementary Fig 4, related to Fig 3. ***Tet2*** **gene transcription analysis in the novel subpopulation of β cells (Btm) vs normal β cells (Top).** Top and Btm β cells were sorted from 10-wk-old NOD mice based on Zinc and TMRE staining. RNA was recovered and the *Tet2* transcription level was measured by qRT-PCR. Results are mean ± SD of 3 sortings, n=3 mice each sorting. Statistical analysis was performed using two-tailed unpaired T-test.

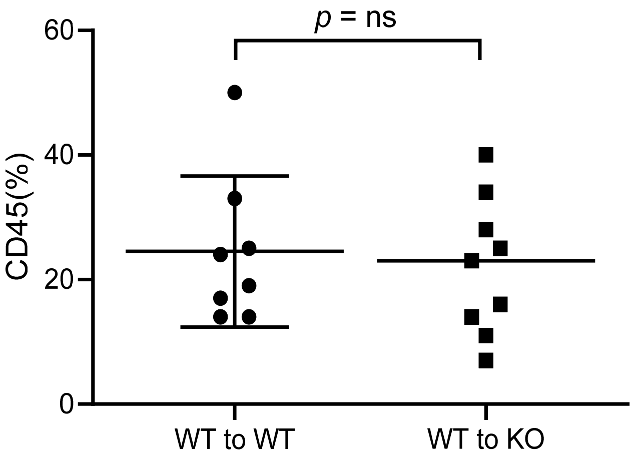

Supplementary Fig 5, related to Fig 4. **The level of CD45+ infiltrates in islets from *Tet2*$^{-/-}$ (Tet2-/-) vs *Tet2*$^{+/+}$ (WT) BMT recipients from WT NOD mice**. 8 weeks post-BMT, CD45+ % of total cells in the islets was analyzed by flow and collected for transcriptional profiling by Nanostring while β cells (Zinc+TMRE+) were enriched for RNAseq as well as ATACseq analysis. One dot represents one recipient. Data are mean ± SD. Statistical analysis was performed using two-tailed unpaired T-test.

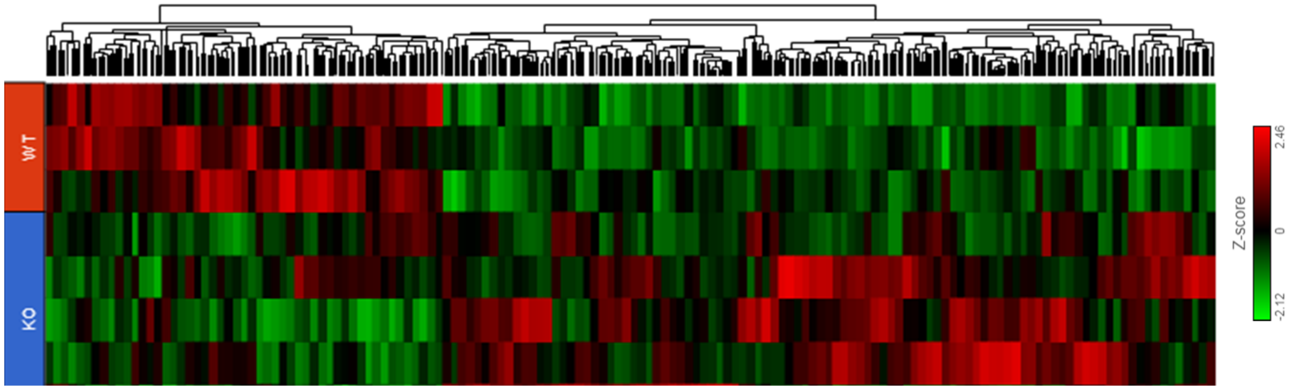

Supplementary Fig 6, related to Fig 5. Heat map generated from the 333 genes that are differently expressed between β cells from *Tet2*$^{-/-}$ vs *Tet2*$^{+/+}$ BMT recipients from *Tet2*$^{+/+}$ NOD by bulk RNASEQ analysis (FDR<0.05).

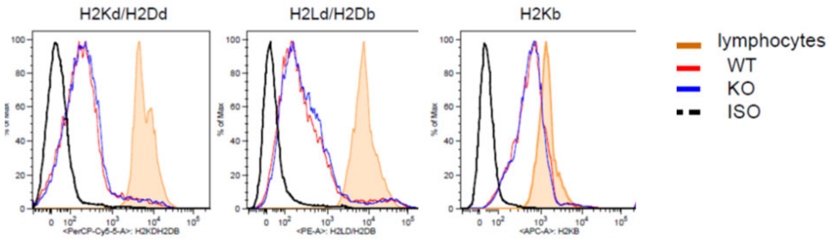

Supplementary Fig 7, related to Fig 5. **The level of MHCI components on β cells from Tet2-/- vs Tet2+/+ B6 mice**

Histogram showing the surface level of MHCI components on β cells from B6 Tet2+/+ and Tet2-/- mice analyzed by FACS. Tet2+/+ lymphocytes were included as positive control vs Isotype negative control (ISO). Data represent 3 experiments and a pair of Tet2-/- and Tet2+/+ mice each experiment.

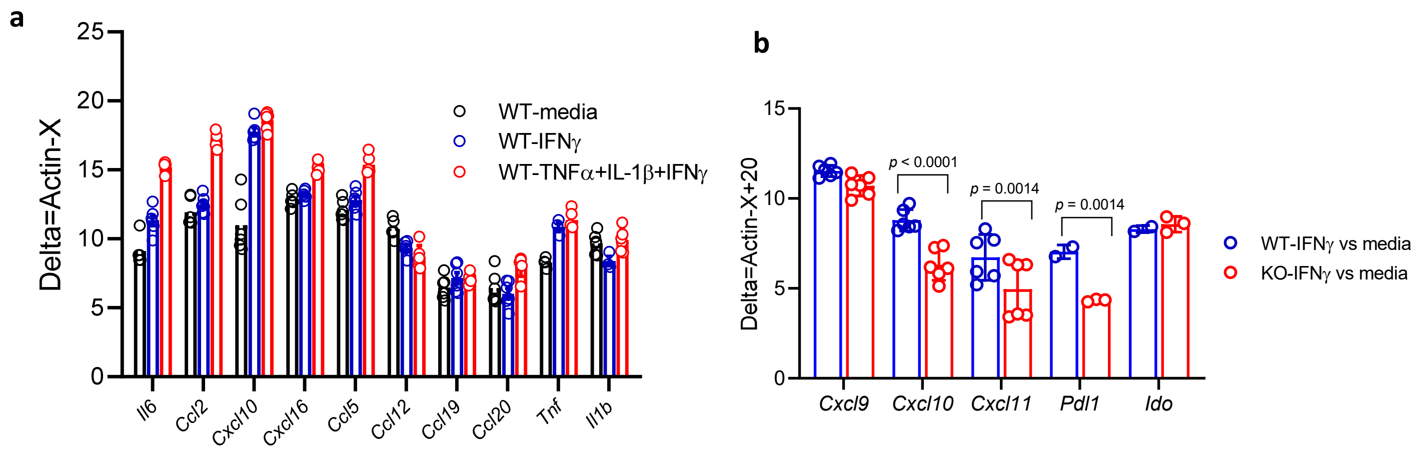

Supplementary Fig 8, related to Fig 6. **In vitro cytokine response from Tet2-/- vs Tet2+/+ islets**. (a) The inducible cytokines and chemokines in islets following cytokine culture. Islets from B6 mice were cultured in the presence of either IFNγ or TNFα+IL-1β+IFNγ for 24 hrs and the transcription profile of candidate chemokine and cytokine species was determined by qRT-PCR. Data are mean ± SD of 4 independent experiment, 4 mice per experiment. (b) Islets from 6-8-week-old B6 mice were cultured in the presence of 10ng/ml IFNγ for 24 hrs and the induction of IFNγ responsive genes as shown was analyzed and compared between Tet2-/- and Tet2+/+ samples. Data are mean ± SD of 3-6 independent experiment, 6 Tet2+/+ and Tet2-/- mice were used each experiment. Statistical analyses were performed using two-tailed unpaired t-tests without correction for multiple comparisons.

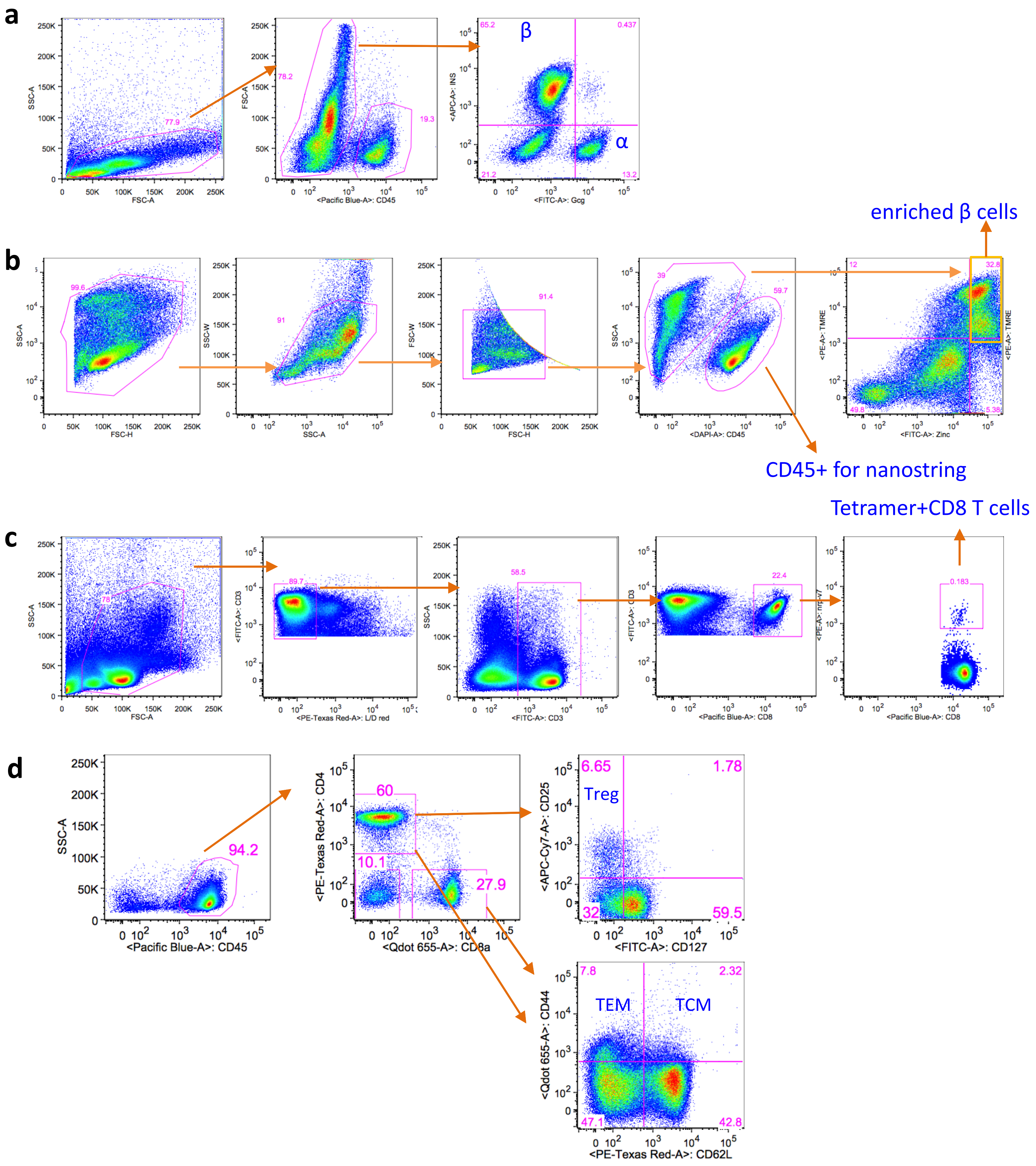

Supplementary Fig 9. **FACS sequential gating strategies for the study.** The sequential gating strategies are representatively shown in **a** for α and β cells in the islets from a 8-wk-old NOD female, as seen in Figures 1d, 3b, 3f-3g and supplementary Figure 1c-e; **b** for enriched β cells that are TMRE+Zinc+, as seen in Figures 1b, 4a, 5a-5b; **c** for Gad reactive CD8T cells via nrvp7 tetramer staining, as seen in Figures 3c and 4d; **d** for CD4/CD8 TEM(CD44+CD62L-) and TCM (CD44+CD62L+) as well as Treg(CD4+CD25+CD127low) populations from pancreatic lymph nodes, as seen in Figures 4b-4c . Data represent at least 5 individual experiments.
